# Mannose-Binding Lectin Deficiency Is Associated With Smaller Infarction Size and Favorable Outcome in Ischemic Stroke Patients

**DOI:** 10.1371/journal.pone.0021338

**Published:** 2011-06-21

**Authors:** Michael Osthoff, Mira Katan, Felix Fluri, Philipp Schuetz, Roland Bingisser, Ludwig Kappos, Andreas J. Steck, Stefan T. Engelter, Beat Mueller, Mirjam Christ-Crain, Marten Trendelenburg

**Affiliations:** 1 Laboratory of Clinical Immunology, Department of Biomedicine and Clinic for Internal Medicine, University Hospital Basel, Basel, Switzerland; 2 Department of Endocrinology, University Hospital Basel, Basel, Switzerland; 3 Department of Neurology, University Hospital Basel, Basel, Switzerland; 4 Department of Emergency Medicine, University Hospital Basel, Basel, Switzerland; 5 Medical University Clinic, Kantonsspital Aarau, Aarau, Switzerland; 6 Harvard School of Public Health, Boston, Massachusetts, United States of America; University of Muenster, Germany

## Abstract

**Background:**

The Mannose-binding lectin (MBL) pathway of complement plays a pivotal role in the pathogenesis of ischemia/reperfusion (I/R) injury after experimental ischemic stroke. As comparable data in human ischemic stroke are limited, we investigated in more detail the association of MBL deficiency with infarction volume and functional outcome in a large cohort of patients receiving intravenous thrombolysis or conservative treatment.

**Methodology/Principal Findings:**

In a post hoc analysis of a prospective cohort study, admission MBL concentrations were determined in 353 consecutive patients with an acute ischemic stroke of whom 287 and 66 patients received conservative and thrombolytic treatment, respectively. Stroke severity, infarction volume, and functional outcome were studied in relation to MBL concentrations at presentation to the emergency department. MBL levels on admission were not influenced by the time from symptom onset to presentation (p = 0.53). In the conservative treatment group patients with mild strokes at presentation, small infarction volumes or favorable outcomes after three months demonstrated 1.5 to 2.6-fold lower median MBL levels (p = 0.025, p = 0.0027 and p = 0.046, respectively) compared to patients with more severe strokes. Moreover, MBL deficient patients (<100 ng/ml) were subject to a considerably decreased risk of an unfavorable outcome three months after ischemic stroke (adjusted odds ratio 0.38, p<0.05) and showed smaller lesion volumes (mean size 0.6 vs. 18.4 ml, p = 0.0025). In contrast, no association of MBL concentration with infarction volume or functional outcome was found in the thrombolysis group. However, the small sample size limits the significance of this observation.

**Conclusions:**

MBL deficiency is associated with smaller cerebral infarcts and favorable outcome in patients receiving conservative treatment. Our data suggest an important role of the lectin pathway in the pathophysiology of cerebral I/R injury and might pave the way for new therapeutic interventions.

## Introduction

Complement mannose-binding lectin (MBL), a member of the collectin subfamily of C-type lectins, is a key component of innate immunity [1]. Besides its important contribution to host defense against pathogens, MBL is also involved in the binding and removal of apoptotic cells leading to complement activation in an antibody- and complement C1q independent manner [2], [3]. In humans polymorphisms within the coding and promoter regions of the MBL2 gene lead to functional MBL deficiency in up to one third of the Caucasian population characterized by reduced levels of circulating functional MBL multimers [4]. During lifetime individual MBL serum levels remain remarkably stable highlighting the dominating influence of genetics as compared to environmental factors. Even during acute inflammation, low MBL serum concentrations are predictive for moderate to severe deficiency [5]. However, there is considerable interindividual MBL level variability (up to ten-fold) in people with the same genotype.

Numerous studies have acknowledged the deleterious effects of complement activation in the pathogenesis of ischemia/reperfusion (I/R) injury (reviewed in [6]) via free-radical production, activation of the coagulation cascade and direct tissue damage. In particular, several lines of evidence from murine models, one human experimental model, and one clinical trial emphasize a central and active role for complement MBL in aggravating tissue damage after I/R injury of the heart [7], [8], [9], [10], kidney [11], gut [12], [13], and skeletal muscle [14]. With respect to cerebral I/R injury results from two recent experimental studies demonstrate that MBL deficiency or inhibition leads to diminished complement C3 deposition and neutrophil influx into the affected brain region and is associated with smaller infarct volumes and better functional outcomes in mice [15], [16]. In humans comparable data are scarce. Activation of the complement system has been confirmed in three clinical studies after acute ischemic stroke [17], [18], [19]. Regarding the effect of MBL, Cervera et al. could demonstrate a significant association of genetically defined MBL deficiency with favorable outcome after three months, though in a small cohort that included a considerable number (19%) of hemorrhagic stroke patients [15]. Moreover, the impact of MBL deficiency on the outcome after thrombolytic treatment was not analyzed. Thrombolytic agents cause a striking systemic activation of the complement system [20], but data on the role of MBL in cerebral I/R injury after thrombolysis is lacking. Hence, the aim of this study was to investigate the impact of functional MBL deficiency in human acute ischemic stroke in a prospective cohort of consecutive patients receiving intravenous thrombolysis or conservative treatment according to the European Guidelines [21].

## Methods

### Ethics Statement

The study had been approved by the local ethical committee at the University Hospital of Basel and all participants or their representative gave written informed consent for the study.

### Participants

We conducted a post hoc analysis of a previously published, prospective cohort study at the University Hospital Basel, Basel, Switzerland consisting of 359 patients presenting to the emergency department with an acute ischemic stroke within 72 hours after symptom onset between November 2006 and November 2007. Patients were classified as either receiving conservative treatment (including monitoring of blood pressure, oxygen saturation, serum glucose, temperature and neurological symptoms, early mobilization, prevention of complications, and treatment of hypoxia, hyperglycemia, pyrexia and dehydration) or intravenous thrombolysis according to the European Guidelines [21]. A complete description of the study including information about clinical work-up, recorded clinical variables, blood-sampling and neuroimaging has been reported previously [22].

### Definitions of endpoints

The primary endpoint for this analysis was favorable functional outcome (score 0 or 1) on the modified Rankin scale (mRS) within 90 days after admission. Secondary endpoints included functional status as assessed by the Barthel Index (BI) at day 90 (favorable outcome was defined as BI> = 95%), death from any cause within a 90-day follow-up, severity of stroke as assessed by the National Institutes of Health Stroke Scale (NIHSS) score on admission, and infarction volume assessed by magnetic resonance imaging (MRI) during initial work-up (categorized according to Katan and colleagues [22]). MRI scans were available in 157/297 and 42/66 patients receiving conservative and thrombolytic treatment, respectively.

### MBL level analysis

For the MBL analysis serum samples drawn immediately on admission to our emergency department (within 0–72 hours after symptom onset) prior to any diagnostic imaging or thrombolytic treatment were available from 353 of 359 patients, of whom 287 and 66 individuals received conservative and intravenous (i.v.) thrombolytic treatment, respectively. For the i.v. treatment group we analyzed additional samples 24 and 72 hours after admission which were available from 56 patients. Quantification of functional MBL was performed by an investigator blinded to any patient data using a commercially available Sandwich-ELISA Kit (*MBL Oligomer ELISA KIT 029*, Lucerna Chem, Luzern, Switzerland) as described previously [23]. Since the definition of functional MBL deficiency is likely to be dependent on the clinical setting, we first analyzed the predefined endpoints in relation to MBL levels. Only in a secondary analysis functional MBL deficiency was defined as serum levels ≤100 ng/ml, as this cut-off has been shown to discriminate reasonably well between individuals with or without homozygosity for MBL variant alleles [24] and to be clinical relevant in human cardiac IR injury [8]. As our study was not powered to identify significant differences in mortality with regard to the predefined MBL cut-off, patients were stratified according to MBL level tertiles in the analysis of Kaplan-Meier survival curves.

### Statistical analysis

Differences in patient characteristics and outcome measures according to treatment and MBL serostatus were analyzed using the Fisher's exact test or the Mann-Whitney-U-Test where appropriate. Due to the non-*Gaussian* distribution of human MBL levels two-group comparisons of MBL concentrations were performed using a Mann-Whitney-U-test, whereas for multigroup comparison a Kruskal-Wallis one-way analysis of variance or Friedman test was applied where appropriate. Dunn's post test was used to correct *p*-values for multiple comparisons. Mortality was analyzed by the log-rank test. Kaplan-Meier estimates were plotted over the observation period of 90 days.

Stepwise logistic regression models were used to estimate the association of MBL levels on predefined endpoints in multivariate analyses after adjustment for covariables being present *prior* to the ischemic event with univariate *p* values less than 0.1. Covariables tested in univariate analyses included patient age, sex, vital parameters and Charlson comorbidity index on admission, history of vascular risk factors, time from symptom onset to admission, and body mass index. All testing was two-tailed, and *p* values less than 0.05 were considered to be statistically significant.

## Results

### Patients' characteristics

Admission serum samples were available from 353 of 359 consecutive patients with an acute ischemic stroke and a complete follow-up, of whom 287 and 66 patients underwent conservative treatment and intravenous thrombolysis, respectively. The baseline and outcome characteristics of the whole study cohort and classified according to the therapeutic intervention are summarized in Table 1. In summary, patients in the conservative treatment group were older (median age of 76 (interquartile range (IQR) 66–83) vs. 71 (IQR 57–70), p<0.001), suffered from more comorbidities (median Charlson Index of 1 (IQR 0–2) vs. 0 (IQR 0–1), p = 0.002), and presented with a less severe stroke (median NIHSS score of 4 (IQR 2–8) vs. 12 (IQR 7–19), p<0.001), whereas unadjusted outcomes after 90 days were similar (data not shown).

**Table 1:** Baseline characteristics of the study population.

Baseline characteristics	All(n = 353)	Conservative treatment(n = 287)	Thrombolysis(n = 66)
Age (median, IQR)	75 (63–82)	76 (66–83)	71 (57–79)
Female sex, n (%)	145 (41)	123 (43)	22 (33)
MBL in ng/ml (median, IQR)	1482 (411–2675)	1461 (408–2857)	1498 (475–2250)
NIHSS on admission (median, IQR)	5 (2–10)	4 (2–8)	12 (7–19)
Charlson index (median, IQR)	1 (0–2)	1 (0–2)	0 (0–1)
Vascular risk factors			
Hypertension, n (%)	269 (76)	220 (77)	49 (74)
D.m., n (%)	69 (20)	61 (21)	8 (12)
Coronary heart disease, n (%)	88 (25)	74 (26)	14 (21)
Hypercholesterolemia, n (%)	91 (26)	76 (26)	15 (23)
Atrial fibrillation, n (%)	74 (21)	63 (22)	11 (17)
Prior stroke, n (%)	86 (24)	74 (26)	12 (18)
Family history of stroke and/or myocardial infarction, n (%)	105 (30)	86 (30)	19 (29)
Stroke etiology *			
Small-vessel occlusion, n (%)	55 (15)	51 (18)	4 (6)
Large-artery atherosclerosis, n (%)	64 (18)	53 (18)	11 (17)
Cardioembolism, n (%)	130 (37)	100 (35)	30 (45)
Other - determined, n (%)	17 (5)	11 (4)	6 (9)
Undetermined, n (%)	87 (25)	72 (25)	15 (23)
Stroke syndrome			
TACS, n (%)	40 (11)	20 (7)	20 (30)
PACS , n (%)	160 (45)	125 (44)	35 (53)
LACS , n (%)	72 (20)	67 (23)	5 (8)
POCS , n (%)	81 (23)	75 (26)	6 (9)
Outcome measures			
mRS day 90 (median, IQR)	2 (1–4)	2 (1–4)	2 (1–5)
mRS 0–1 day 90, n (%)	161 (46)	135 (47)	26 (40)
Death, n (%)	42 (12)	32 (11)	10 (15)

Abbreviations: mRS = modified Rankin Scale; IQR = interquartile range; NIHSS = National Institutes of Health Stroke Scale; TACS = total anterior circulation syndrome; PACS = partial anterior circulation syndrome; LACS = lacunar syndrome; POCS = posterior circulation syndrome; D.m. = Diabetes mellitus;

*Stroke etiology according to the TOAST classification [39].

### Mannose-binding lectin serum levels

On admission the median MBL serum level of all patients included in this study was 1482 ng/ml (IQR 411–2675 ng/ml), and 37 of 353 (10%) individuals showed levels below 100 ng/ml, thereby closely resembling frequency distributions observed in the general population [4], [24]. Furthermore, initial MBL levels were similar in patients presenting to our emergency department within 0–3 (median 1502 ng/ml), 3–12 (median 1388 ng/ml), 12–24 (median 1768 ng/ml) and 24–72 hours (median 1607 ng/ml) after symptom onset (p = 0.53), as was the case in the two treatment groups. Sequential analysis of MBL serum levels in the thrombolysis group revealed stable MBL levels during the first 24 hours after administration of thrombolytic agents (admission: median 1500 (IQR 490–2250) ng/ml; after 24 hours: median 1610 (IQR 459–2259) ng/ml). However, a significant increase in MBL levels occurred after five days (median 2167 (IQR 548–3281) ng/ml, p<0.001 for the comparison of admission vs. day 5), reflecting a mild acute phase reaction. As expected from previous studies, this increase was only observed in MBL sufficient patients, whereas MBL levels remained constant in MBL deficient patients [5].

### Conservative treatment group

#### Association with functional outcome and death after ischemic stroke

**Figure 1:**
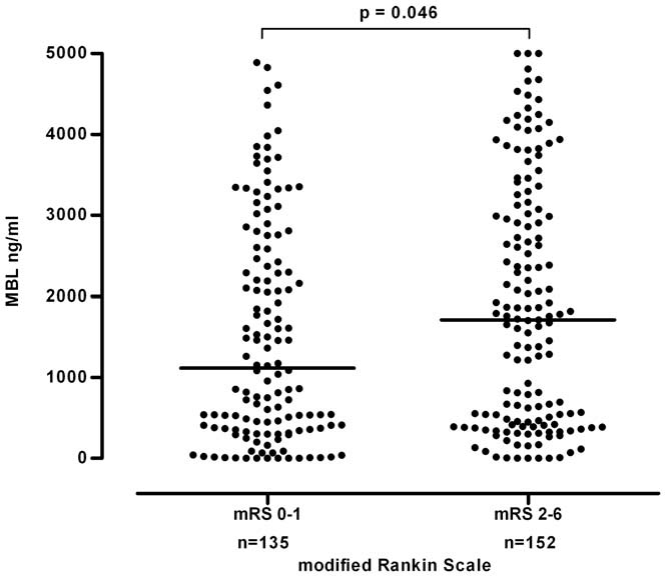
Association of functional outcome with serum MBL levels. Conservative treatment group: differences in MBL serum concentrations according to the functional outcome as assessed by the modified Rankin Scale 90 days after presentation. Horizontal lines represent medians.

Uni- and multivariate analysis of the primary endpoint showed that MBL levels were significantly associated with functional outcome after three months in this treatment group: Patients with a complete recovery (i.e., a mRS of 0 or 1) had lower MBL levels than individuals with an unfavorable outcome (median MBL level 1116 (IQR 370–2524) vs. 1713 (IQR 420–3018) ng/ml, p = 0.046, Figure 1). After adjusting for potential confounders being present *prior* to the ischemic event MBL levels remained independently associated with an unfavorable outcome three months after ischemic stroke (OR 1.23, 95% CI 1.02–1.48, for every 1000 ng/ml increase, p = 0.036) in addition to age (Table 2).

**Table 2:** Multivariate Analysis: predictors of an unfavorable outcome (defined as a score of 2–6 on the modified Rankin Scale) three months after ischemic stroke.

	Multivariate Analysis		
Predictor	OR	95% CI	P-value
MBL levels (increase per 1000 ng/ml)	1.23	1.02–1.48	0.036
Age (increase per year)	1.05	1.03–1.07	<0.0001
History of prior stroke	1.76	0.96–3.21	0.066
Charlson Comorbidity index (increase per unit)	1.19	0.99–1.42	0.066

Abbreviations: OR = odds ratio; CI = confidence interval.

To confirm the influence of MBL levels on functional recovery after ischemic stroke a second outcome measure (Barthel Index) was analyzed. A strong trend towards higher MBL levels in patients with an unfavorable recovery within 90 days after admission (defined as a BI score of > = 95%) could be observed as compared to individuals with a favorable outcome (median MBL levels 1754 vs. 1270 ng/ml, p = 0⋅06), which was significant in the multivariate analysis (OR 1.26, 95% CI 1.04–1.54, for every 1000 ng/ml increase, p = 0.02).

**Figure 2:**
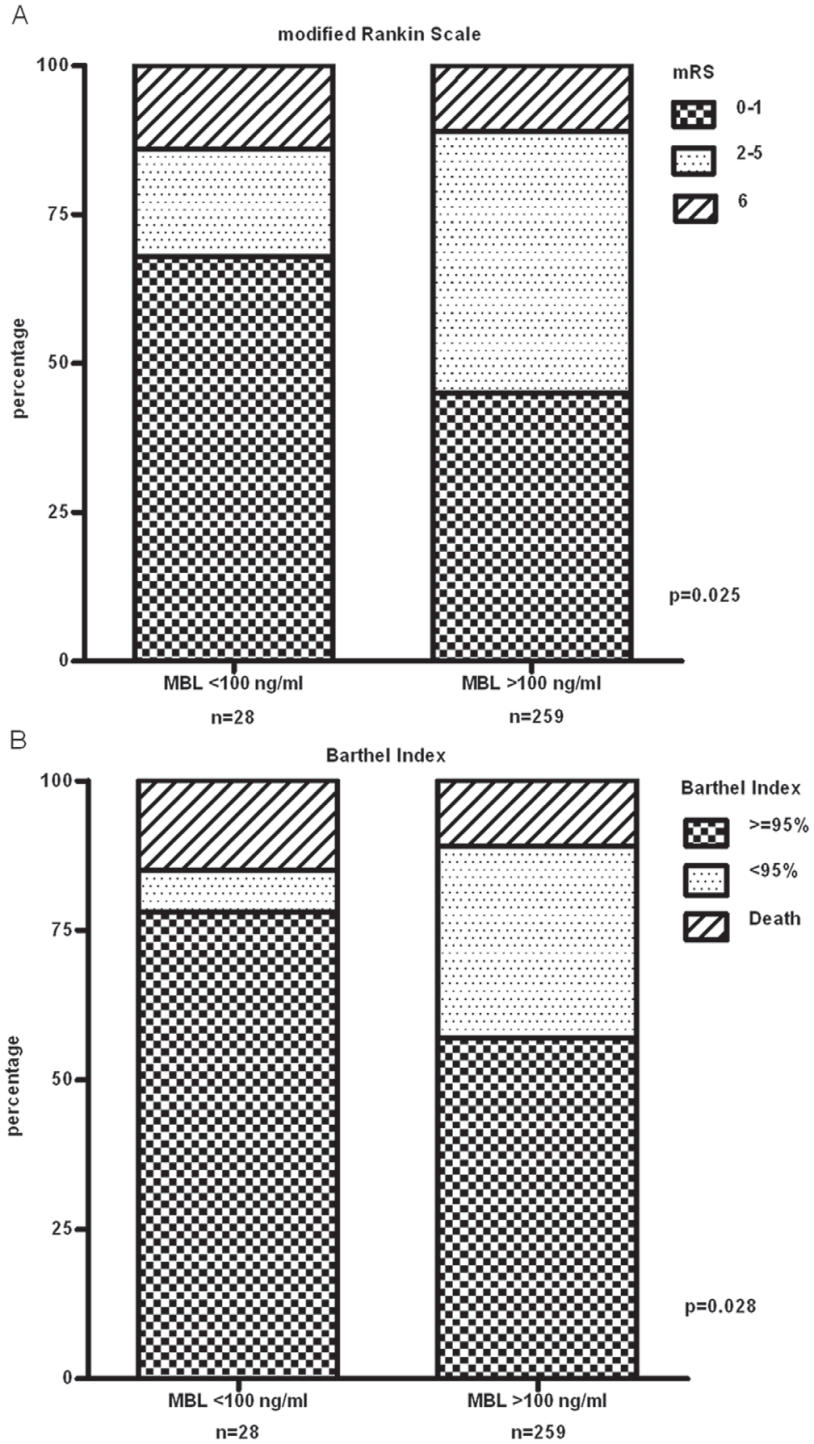
Association of MBL deficiency with functional recovery. Conservative treatment group: association of functional MBL deficiency (MBL<100 ng/ml) with functional recovery three months after ischemic stroke as assessed by (A) the modified Rankin Scale and (B) the Barthel Index.

The conservative treatment group consisted of 28/287 (9.8%) patients with MBL levels below 100 ng/ml, a cut-off generally accepted for the definition of functional MBL deficiency. Baseline characteristics were similar in MBL deficient patients as compared to patients with MBL levels ≥100 ng/ml with the exception of a history of hypertension (15/28 vs. 205/259, p = 0.004). In this MBL deficient group there were 19 patients (68%) with a favorable recovery (i.e., a mRS score of 0 or 1) three months after admission, five (18%) with an unfavorable outcome (mRS score of 2–5) and four (14%) individuals who died (mRS score of 6), as compared to 116 (45%), 115 (44%) and 28 (11%) corresponding patients with MBL levels above 100 ng/ml (p = 0.025, Figure 2A). In a multivariate analysis (including history of hypertension as a cofactor) MBL deficient patients were subject to an almost 3-fold decreased risk to suffer from significant disabilities or to die within three months after ischemic stroke (OR 0.38, 95% CI 0.14–0.98, p = 0.046). Besides absence of MBL deficiency, only older age (OR 1.05, 95% CI 1.02–1.07, for every year increase, p<0.0001) and more comorbidities (OR 1.22, 95% CI 1.02–1.46, for every unit increase in the Charlson Comorbidity index, p = 0.03) were independent predictors of an unfavorable outcome after three months. Analysis of the secondary outcome measure, the Barthel Index further underscored the beneficial effect of MBL deficiency in ischemic stroke (p = 0.028, Figure 2B).

For the association with mortality alone Kaplan-Meier survival curves were calculated stratified by MBL tertiles. The risk for death within three months after admission was lowest in patients in the first tertile (MBL<542 ng/ml; survival rate 92±2.8%), intermediate in the second (MBL 542–2288 ng/ml; survival rate 89±3.1%) and highest in the third tertile (MBL>2288 ng/ml, survival rate 86±3.5%). However, these differences did not reach statistical significance (p = 0.26 for the comparison of the first vs. third tertile; data not shown).

#### Association with severity and size of stroke

**Figure 3:**
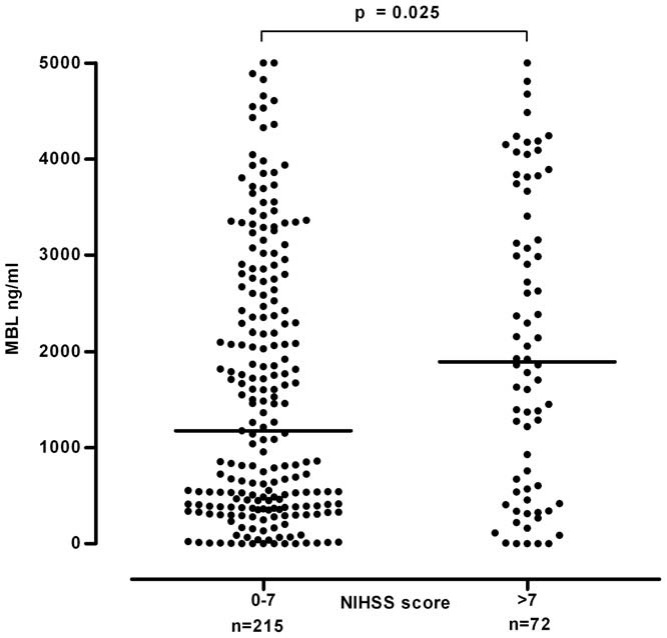
Association of stroke severity with serum MBL levels. Conservative treatment group: differences in MBL serum concentrations according to stroke severity as assessed by the NIHSS at presentation. Horizontal lines represent medians.

**Figure 4:**
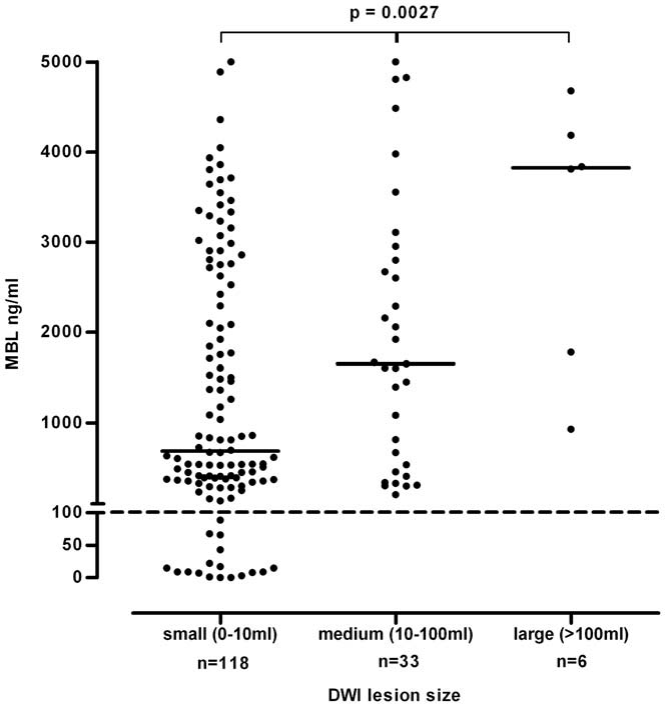
Association of lesion size with serum MBL levels. Conservative treatment group: differences in MBL serum concentrations according to infarction volume on MRI scan (subgroup analysis). Horizontal lines represent medians. Horizontal dashed line corresponds to the cut-off of <100 ng/ml representing MBL deficient patients. Abbreviation: DWI = Diffusion-weighted imaging.

Patients with a mild stroke at presentation (defined as a NIHSS score of ≤7 [25], [26]) showed significantly lower MBL levels than individuals with intermediate or high (8–14 and >14, respectively) NIHSS scores (median MBL level 1176 (IQR 386–2640) vs. 1893 (IQR 555–3536) ng/ml, p = 0.025, Figure 3). Analysis of a subgroup of patients (n = 157 in whom MRI as part of the initial work-up was available revealed a striking association between lesion size and MBL serum levels. Median MBL levels were lowest in patients with a small (<10 ml) lesion (685 (IQR 255–2577) ng/ml) as compared to patients with medium (10–100 ml) and large (>100 ml) infarction volumes (1653 (IQR 497–2877) ng/ml and 3826 (IQR 1356–4433) ng/ml, respectively. p = 0.0027, Figure 4).

Furthermore, MBL deficient patients (<100 ng/ml) showed significantly smaller infarctions as compared to MBL sufficient patients (mean size 0.6 (SD 0.8) vs. 18.4 (SD 43.6) ml, p = 0.0025). Notably, none of the MBL deficient patients experienced a medium or large cerebral lesion (volume of >10 ml) as compared to 39 of 140 (28%) patients with MBL levels above 100 ng/ml (p = 0.0073).

After adjustment for covariables being present *prior* to the ischemic event only MBL levels remained independently associated with lesion size on MRI: The average infarction volume increased by 7.3 ml for every 1000 ng/ml increase in MBL serum levels (standard error 2.32, 95% CI 2.9–12.2, p = 0.002).

### Thrombolysis treatment group

In contrast to the conservatively treated group MBL levels did not influence any of the above mentioned outcome measures in patients receiving thrombolytic agents after admission. Notably, median MBL levels were similar in patients with a less severe (NIHSS score <7) vs. a severe stroke (NIHSS score >7) at presentation (1508 vs. 1488 ng/ml, p = 0.96), in patients with small vs. medium/large stroke lesions on MRI (1315 vs. 1482 ng/ml, p = 0.66), in patients with a complete recovery vs. an unfavorable outcome (1325 vs. 1680, p = 0.24) and in patients surviving vs. dying during a 90-day follow-up (1620 vs. 1341 ng/ml, p = 0.5). This holds also true for the cut-off of <100 ng/ml, representing MBL deficient patients.

### Pooled analysis

If all study participants (thrombolysis and conservative treatment group) were analyzed together, MBL levels were still independently associated with an unfavorable outcome three months after ischemic stroke (OR 1.23, 95% CI 1.03–1.46, for every 1000 ng/ml increase, p = 0.019) in addition to only age (OR 1.03, 95% CI 1.02–1.05, for every year increase, p<0.0001). Furthermore, the association of low MBL levels with smaller infarction size persisted in the pooled analysis (data not shown).

## Discussion

Several lines of evidence have emphasized the pivotal role of the complement system in general and the MBL pathway in particular in the pathogenesis of I/R injury, including ischemic stroke [15], [16], [17], [18], [19]. In the present study, we demonstrate that functional MBL deficiency is associated with smaller cerebral lesion volumes and favorable outcomes in patients with conservative treatment after acute ischemic stroke but not in patients receiving i.v. thrombolysis.

These results are in line with data from Cervera et al. who demonstrated a significant association of genetically defined MBL deficiency with favorable outcome albeit in a smaller mixed hemorrhagic and ischemic stroke cohort [15]. By analyzing infarction size on MRI and comparing outcome measures in a conservative vs. thrombolytic treatment cohort we highlight in more detail the potential pathogenetic relevance of the MBL pathway after human ischemic stroke. In the conservative treatment group MBL deficiency seems to considerably restrict propagation of damage after ischemic stroke. Patients with MBL levels <100 ng/ml were almost three times more likely to recover without significant disabilities within three months after admission when compared to MBL sufficient patients. This protective effect was even more pronounced when analyzing the association of MBL levels with infarction volumes on MRI in a subgroup of patients. Compared to patients with a small cerebral lesion median MBL serum concentration was more than two-fold and five-fold higher in patients with medium and large infarction volumes, respectively. In particular, functionally MBL deficient patients invariably experienced small cerebral lesions. This is well in line with experimental data using either MBL-deficient mice or an inhibitor of the MBL pathway [15], [16].

Notably, the magnitude of association was more evident in the analysis of early infarction volumes than of functional outcome. Hence, the role of MBL seems to be most prominent in the early phase of ischemic stroke, whereas functional recovery might not only be influenced by lesion size and stroke severity (as assessed by the NIHSS) but several other factors such as age, localization of infarct, secondary complications, and early initiation of rehabilitation services [28]. Alternatively, data from Rahpeyma Y et al. suggest that complement activation in the brain may not only exacerbate tissue damage in the acute setting (lesion size) but on the other hand also promote neurogenesis and regeneration after ischemia in the long-term (functional outcome) [27].

A significant influence of MBL deficiency on *mortality* after ischemic stroke was not evident in our study. Considering that there were only 32 events in the conservatively treated group (11%), the study was not powered to analyze smaller differences between MBL sufficient and deficient patients. Furthermore, mortality after stroke might be more affected by preexisting comorbidities and infections than MBL status.

Interestingly, we did not observe a similar protective effect of MBL deficiency in patients receiving i.v. thrombolysis, which in theory should more closely mimic experimental I/R injury than conservative treatment. Several reasons might account for this important difference. First, our thrombolysis group was rather small and only powered to detect substantial differences in outcome, yet results were consistent over all endpoints analyzed. Second, animal as well as human data assessing the effect of MBL deficiency on ischemic stroke outcome after systemic thrombolysis are lacking. Therefore, we a priori chose to separately analyze patients receiving systemic thrombolysis vs. conservative treatment. In theory, massive systemic complement activation occurring after thrombolysis might have masked or offset any favorable effect of MBL deficiency that is usually evident after experimental cerebral I/R injury [20]. On the other hand, the benefit of a successful thrombolysis might have significantly outweighed any effect of the MBL pathway, e.g. by attenuating inflammatory reaction after ischemic stroke which is only partly mediated by the MBL pathway. Audebert et al. found that successful thrombolysis was associated with subsequently lower C-reactive protein (CRP) and white blood count levels [29]. Moreover, CRP levels were not predictive of functional outcome in a study of patients receiving thrombolysis [30] in contrast to several studies involving exclusively or mainly conservatively treated patients [31], [32], [33], [34], thereby further underscoring the influence of thrombolysis on inflammation after stroke. In conclusion, additional prospective trials are warranted to further investigate the effect of the MBL pathway in patients undergoing thrombolysis.

Recently, recombinant human C1-Inhibitor has been shown to function as a powerful inhibitor of MBL and its detrimental cerebral effects in a mouse model of cerebral I/R injury [16]. By demonstrating smaller lesion volume and improved functional outcome in MBL deficient patients the present study draws further attention to MBL as a promising target for reducing cerebral I/R injury and supports the investigation of functional MBL inhibition in human ischemic stroke, especially in patients who do not qualify for thrombolytic treatment.

Despite the precisely characterized cohort the present prospective observation study has limitations including the post hoc analysis of MBL serum levels. Furthermore, MRI testing was not available for all patients in both groups, which may have biased results. Yet, analysis of functional outcome in patients with MRI data revealed similar effect of MBL deficiency as compared to the whole cohort (data not shown). Regarding the thrombolysis group the small sample size limits the significance of the observed data. At first sight the fact that *MBL2* genotypes were not determined could appear as a limitation. However, MBL serum levels show little variation throughout life. In particular, MBL levels have never been shown to strongly fluctuate during acute diseases, and MBL levels in our patients were not influenced by the interval from symptom onset to presentation and blood sampling in our emergency department. Therefore, measurement of MBL serum levels by ELISA allows reliable quantification of the functional activity of the MBL pathway *in vivo* in this setting [35]. When evaluating associations with diseases measurement of MBL serum levels might in fact represent a more sensitive approach than determination of genotypes as individuals with the same genotype may vary up to tenfold in MBL serum levels [5], [36], [37]. Moreover, *MBL2* genotyping cannot account for the significant changes of MBL serum levels induced by thyroid dysfunction [38].

In conclusion, functional MBL deficiency was associated with smaller infarction volume and favorable functional outcome in patients receiving conservative treatment after acute ischemic stroke. These findings support the concept of a significant contribution of the MBL pathway to cerebral tissue injury in human ischemic stroke. Thus, transient and early blockade of MBL or inhibition of the lectin complement pathway may represent a therapeutically promising strategy for reducing I/R associated cerebral damage.
